# Advanced biomaterials and virtual reality for interventions in rare episodic cluster headache mimicking SUNCT syndrome: emerging directions in precision pain management

**DOI:** 10.1097/MS9.0000000000003782

**Published:** 2025-08-28

**Authors:** Zeeshan Ahmed, Muneeb Saifullah, Maliha Khalid, Muhammad Talha, Aminath Waafira

**Affiliations:** a King Edward Medical University, Lahore, Pakistan; b Jinnah Sindh Medical University, Karachi, Pakistan; c School of Medicine, The Maldives National University, Malé, Maldives

**Keywords:** biomaterials, cluster headache, precision pain management, SUNCT syndrome

## Abstract

Rare episodic cluster headache mimicking SUNCT syndrome presents a unique clinical challenge due to overlapping trigeminal autonomic features and limited therapeutic efficacy of conventional interventions. Recent innovations in advanced biomaterials, such as poly(L-lactic acid) and PVDF-based piezoelectric scaffolds integrated with decellularized extracellular matrix, offer biocompatible platforms for trigeminal nerve repair and inflammation modulation. Virtual reality (VR) technologies, through immersive pain-modulation environments, enhance neuroplasticity and empower patients in real-time symptom control. Artificial intelligence (AI) algorithms, incorporating multi-omic and wearable sensor data, enable personalized trigger profiling, predictive prevention, and early attack forecasting. CRISPR-based gene-editing strategies targeting pain-modulating genes, such as TACR1, have demonstrated potential in preclinical models for refractory headache management. Integrating these multidisciplinary innovations into clinical frameworks may pave the way for precision pain medicine, improved patient self-management, and enhanced long-term outcomes for those suffering from this debilitating headache disorder.

*To the Editor,*

Cluster headache is a rare and severely painful type of trigeminal autonomic cephalalgia, which remains a substantial clinical challenge, especially with episodic varieties mimicking SUNCT syndrome-short-lasting unilateral neuralgiform headache attacks with conjunctival injection and tearing. Despite much advancement since then in the understanding of trigeminal autonomic pathways, chronic or medically refractory cases remain difficult to manage. The recent studies give a fresh perspective on differentiating the trigeminal autonomic symptoms and intervention targeting those complex headaches^[1]^.

Advanced biomaterial integration has brought new horizons into neural scaffold design for pain pathway repair. The synthetic and hybrid polymer scaffolds, including poly(L-lactic acid) and PVDF-based piezoelectric materials combined with decellularized extracellular matrix, induce favorable microenvironments for regeneration and inflammation modulation of nerve tissue in pain pathways. Such scaffolds are therapeutic agents for restoring disrupted trigeminal nerve function in cluster headache as biocompatible alternatives replacing pharmacological treatments with specificity and tolerability issues^[2]^.

Concurrently, virtual reality (VR) technology is emerging as an adjunct tool for pain management by simulating headache attacks to train patients’ adaptive responses. Escalating studies have indicated that immersive virtual reality, together with standard analgesics, significantly improved pain relief and reduced the need for rescue medications in acute migraine attacks, which, in turn, may favor the use of VR in the management of episodic cluster headaches. These VR environments engage neuroplasticity mechanisms to offer non-invasive behavioral therapies that allow for real-time patient empowerment in symptom-control interventions^[3]^.

Artificial intelligence (AI) is more personalized for headache management by adding multi-omic data to recognize individual trigger profiles. Machine learning models developed into clinical and genomic data improve classification accuracy for headache subtypes, making predictive prevention strategies. Real-time monitoring and early attack forecasting will improve patient outcomes through timely interventions made possible by AI biosensors and wearables^[4,5]^.

CRISPR and other gene-editing mechanisms have limitless promises for the treatment of chronic pain conditions such as cluster headache by targeting pain-modulating genes such as TACR1 that have been found to be relevant to pathophysiology. Using preclinical models, the CRISPR-Cas9 methodology has been used to modulate pain receptor activity with minimal off-target effects and paves the path for future clinical applications in the management of cluster headaches that are refractory. Hence, rigorous clinical trials are needed to establish the long-term effectiveness as well as safety^[6]^. Our work is in line with the TITAN Guidelines on the need for transparency in AI use in healthcare^[7]^.

In summary, the incorporation of precision medicine, advanced biomaterials, and advancing technologies like VR, AI, and CRISPR, offers a strong foundation for the treatment rare episodic cluster headaches that mimic SUNCT syndrome. These multidisciplinary strategies have the power to enhance neural repair, promote self-management of patients, and facilitate genetically informed therapies. This highlights the need to prioritize investment, promote interdisciplinary collaboration, and integrate these innovations into clinical guidelines, thus aiming to improve the quality of life and long-term outcomes for patients with these debilitating headache disorders.

## Data Availability

No data were generated for this manuscript.

## Peer and provenance statement

This article was not commissioned and was externally peer-reviewed.
